# Compartment Syndrome following *Bothrops* Snakebite Leads to Decompressive Fasciotomies

**DOI:** 10.1155/2019/6324569

**Published:** 2019-03-04

**Authors:** Murilo Sérgio Valente-Aguiar, Bruno Gonçalves da Costa e Silva, Teresa Magalhães, Ricardo Jorge Dinis-Oliveira

**Affiliations:** ^1^Department of Public Health and Forensic Sciences, and Medical Education, Faculty of Medicine, University of Porto, Porto, Portugal; ^2^Legal Medical Institute of Porto Velho, Civil Police of the State of Rondônia, Porto Velho, Rondônia, Brazil; ^3^Center for Tropical Medicine (CEMETRON), Porto Velho, Rondônia, Brazil; ^4^IINFACTS-Institute of Research and Advanced Training in Health Sciences and Technologies, Department of Sciences, University Institute of Health Sciences (IUCS), CESPU, CRL, Gandra, Portugal; ^5^UCIBIO-REQUIMTE, Laboratory of Toxicology, Department of Biological Sciences, Faculty of Pharmacy, University of Porto, Porto, Portugal

## Abstract

Snakebite envenoming is a neglected tropical disease with relevant morbidity and mortality. In this report, we illustrate the clinical course of a suspected *Bothrops* snakebite envenoming of a patient that evidenced severe pain, edema, pallor, regional lymphadenopathy, ecchymosis, myonecrosis, and bullous erythema in the right lower limb, specially around the fang marks. The clinical course progressed to compartment syndrome followed with decompressive fasciotomies to reduce pressure within the affected compartment.

## 1. Introduction

Venomous and nonvenomous snakes are worldwide distributed, especially in tropical and subtropical warmer climates since are ectothermic. According to the World Health Organization, snakebite envenoming is a neglected tropical disease representing a public health concern with relevant morbidity and mortality in the developing countries [1–3]. Lance-headed pit vipers (*Bothrops* species) that belong to the family Viperidae are responsible for most of the snake envenoming accidents in Brazil [4]. In this report, we illustrate the clinical course of the *Bothrops* snakebite that progressed to compartment syndrome followed with decompressive fasciotomies to reduce pressure within the affected compartment. Very few case reports have reported postsnakebite compartment syndrome [5–9].

## 2. Case Report

**Figure 1:**
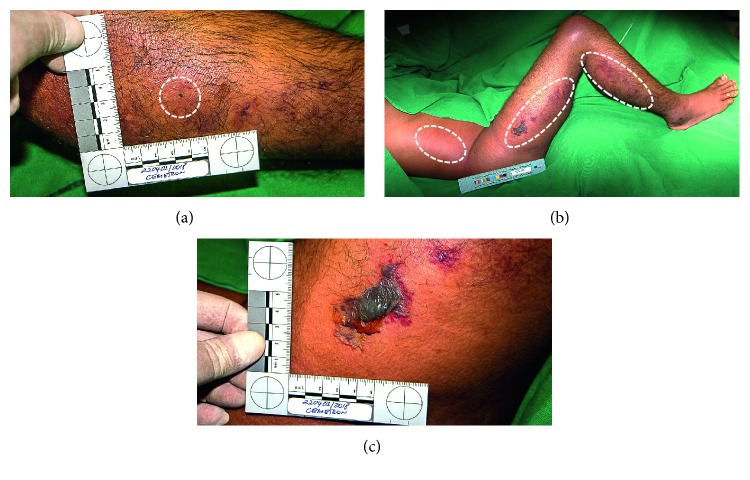
*Bothrops jararaca* (family Viperidae) snakebite on the right leg. (a) Venom entrance. (b, c) Swelling, blistering, and hemorrhage.

**Figure 2:**
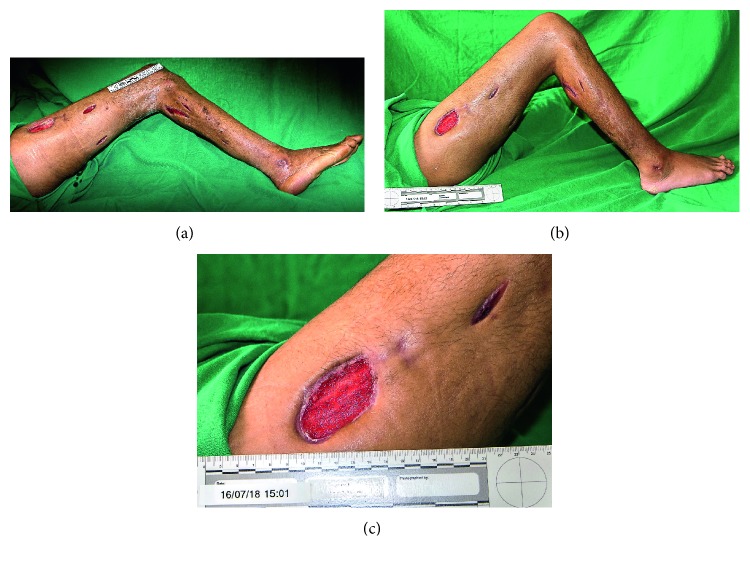
Compartment syndrome and required decompressive fasciotomies to reduce pressure within the affected compartment.

A 28-year-old man sought medical attention reporting that he had been bitten by the snake *Bothrops jararaca*. He presented only pain and a punctate wound on the lateral aspect of the middle third of the right leg and without other signs (Figure 1). Because it was not a characteristic snakebite lesion and a thorn sting was suspected, symptomatic treatment was performed. The physician did not valuate the patient report, and he was then discharged with analgesics. The victim returned to the health unit 5 days after the accident, complaining of severe pain, edema, pallor, regional lymphadenopathy, bruising ecchymosis, myonecrosis, and bullous erythema in the right lower limb, specially around the fang marks (Figure 1). All other clinical data evaluated during the physical examination were normal. At this second admission, white cell count was normal (4.7 × 10^9^/L) but then leukocytosis developed in the second day (14.7 × 10^9^/L) and persisted for approximately 8 days. Creatine kinase levels were very high (3.006 IU × 10^3^/L) at the admission and then begun to decrease during hospital treatment, suggesting recovering of rhabdomyolysis. Thrombocytopaenia (54.300 × 10^9^/L being the lowest registered value); coagulopathy; increase of C-reactive protein (445.28 mg/dL being the highest registered value); sedimentation rate of erythrocyte, *γ*-glutamyltransferase, and lactate dehydrogenase; and a slight alteration in liver transaminase levels were also registered. Acute kidney injury was not observed; creatinine levels were within the normal limits, and serum urea levels were increased (ranging from 60–110 mg/dL during the first 4 days after admission), suggesting in this case increased protein catabolism caused by skeletal muscle injury. He was then treated with 10 vials of antibothropic serum and then 20 vials of a polyvalent antibothropic laquetic antivenin serum. Nevertheless, he progressed to compartment syndrome and required decompressive fasciotomies, aiming to reduce pressure within the affected compartment in order to prevent irreversible sequelae (Figure 2). The victim underwent analgesia with opioids and antibiotic therapy first with ampicillin and sulbactam (for 8 days) and subsequently imipenem with cilastatin and vancomycin (for 21 days) according to the ongoing protocol to control nosocomial infections. He was discharged 71 days after the accident.

## 3. Commentary

Although very uncommon and rarely described with a reported incidence below 6.6% [10], compartment syndrome is a dangerous complication of envenoming by *Bothrops* species leading to myonecrosis, neuropathy, limb amputation, and death, especially if the bite occurred on bare skin, snake's fangs (i.e., modified teeth located in the frontal region of the maxillary bones and connected via a duct to a venom gland) penetrate subfascial compartment, and treatment is delayed [11–14]. In contrast to compartment syndrome pathophysiology as a consequence of trauma, which primarily results from an increased pressure within the affected area, venom-induced compartment syndrome probably involves a direct cytotoxic effect of venom on tissues and a reduced perfusion pressure [11]. In our case, the snake was not captured for identification but is most probably *Bothrops jararaca*, which is the most abundant and clinically relevant species in Brazil [15, 16].

Local toxic effects are mainly due to the action of venom toxins, namely [17, 18], (i) metalloproteinases that hydrolyze key components of the basement membrane of capillaries, particularly type IV collagen, causing weakening of the mechanical stability of microvessels leading to hemorrhage; (ii) phospholipases A_2_ which bind to and disrupt the integrity of the plasma membrane of muscle fibers leading to myonecrosis; and (iii) hyaluronidases that cause extracellular matrix degradation. Besides local effects, venom of *Bothrops* species may also be systemically distributed through the lymphatic system and blood vessels causing systemic alterations, namely, hemorrhage, coagulopathies, acute kidney injury, and neurotoxicity that may progress to respiratory failure and cardiovascular shock [1, 19].

Finally, it is important to highlight that snake venoms are a highly complex protein mixture [20], resulting in a variable biochemical and toxicological profile that determines a wide range of local and systemic clinical signs and symptoms resulting in a challenging diagnosis [1]. After snakebite, the rapid and accurate diagnosis is imperative, and the continuous monitoring of subfascial pressures may be useful in the diagnosis and monitoring of compartment syndrome in order to reduce the risk of soft tissue necrosis and permanent disability [8, 21]. Indeed, it should be remembered that many patients may remain asymptomatic, and compartment syndrome may only develop hours to several days after snakebite, and therefore, the patient should not be discharged without being referred to continuous supervision [22]. Particularly interesting would be the identification of risk factors that combined with clinical presentation could help predict compartment syndrome when patients arrive at the emergency room. Increased levels of white blood cells, probably due to venom proteins and cytokine-induced leukocytosis, and increased activity of aspartate aminotransferase as a consequence of acute hemolysis and necrosis of skeletal muscle are particularly promising predictors [8, 10]. Moreover, surgical decompression of compartment syndrome is most effective and results in fewer complications if it occurs within a “golden period” of 8 to 12 hours postonset and if intracompartment pressure is above 50–55 mmHg [11–14].
